# Global protein expression dataset acquired during isoniazid-induced cytoprotection against H_2_O_2_ challenge in HL-60 cells

**DOI:** 10.1016/j.dib.2016.01.035

**Published:** 2016-01-29

**Authors:** Saifur R. Khan, Argishti Baghdasarian, Richard P. Fahlman, Arno G. Siraki

**Affiliations:** a Faculty of Pharmacy and Pharmaceutical Sciences, University of Alberta, Edmonton, Canada; b Department of Biochemistry, Faculty of Medicine & Dentistry, University of Alberta, Edmonton, Canada; c Department of Oncology, Faculty of Medicine & Dentistry, University of Alberta, Edmonton, Canada

## Abstract

Isoniazid (INH) is one of the first-line anti-tuberculosis drugs. Its effect on oxidative stress, however, is unknown. Here we used a model of oxidative stress by employing glucose/glucose oxidase (GOx), which (based on the availability of glucose and oxygen) is known to produce H_2_O_2_. This reaction induces oxidative stress culminating in necrotic cell death in HL-60 cells (a human promyelocytic leukemia cell line). The changes in protein levels have been quantified using global proteome expression changes through stable isotope labeling by amino acids in cell culture (SILAC) followed by LC–MS/MS analysis. A total of 1459 and 1712 proteins were identified in forward and reverse experiments, respectively. However, only 390 proteins were reproducibly identified in both samples. These 390 proteins were taken into account for further analysis which has been described in “Cytoprotective effect of isoniazid against H_2_O_2_ derived injury in HL-60 cells” [1].

## 1. Specifications table

**Table:** 

Subject area	Biology
More specific subject area	SILAC-based proteomics in a mammalian cell line (HL-60).
Type of data	Proteomic readout tables (MS Excel®)
How data was acquired	Stable Isotope (^13^C_6_) labeling by l-Lysine and l-Arginine in HL-60 cells, run reactions, cell lysis, SDS-PAGE & scanning through LI-COR Odyssey gel scanner, in-gel digestion and LC-MS/MS analysis using nanoflow HPLC (Easy-nLC II, Thermo Scientific) coupled to the LTQ XL-Orbitrap hybrid mass spectrometer (MS) (Thermo Scientific).
Data format	Raw and analyzed data
Experimental factors	Protein expression in HL-60 cells at a 4 h time point
Experimental features	The project profiles and analyzes the expression patterns in INH treated HL-60 cells which had been simultaneously exposed to H_2_O_2_.
Data source location	Edmonton, Alberta, Canada
Data accessibility	Data is within this article

## 2. Value of the data

• SILAC-based proteomics is a suitable method for in vitr*o* study in HL-60 cells.
• The proteomic readout can be applied for pathway analysis (e.g., KEGG, GO, etc.).
• Both qualitative and quantitative aspects of the protein dataset can provide pathway information relating to the specific cellular processes under investigation.
• The dataset can provide a mechanism to study specific proteins involved in cytotoxic/cytoprotective processes.
• These data can be used to carry out further studies into signaling mechanisms by mapping protein signaling pathways.

## 3. Data

The forward SILAC analysis quantified 1459 proteins whereas reverse SILAC analysis quantified 1712 proteins. However, a total of 390 proteins were reproducibly identified and quantified in both forward and reverse reactions using a highly stringent data analysis protocol (see Supplementary data Table, Excel® file). String (version 9.1) analysis was deployed to analyse the co-expression pattern of all significantly expression-changed proteins [1].

## 4. Experimental design, materials & methods

### 4.1 Reactions and treatments in HL-60 cells

To produce oxidative stress, GOx (25 mU/ml of glucose oxidase and 5 mM glucose) was used in the reactions. The rate of H_2_O_2_ generation by GOx was calculated as 2.928±0.072 µmol U^−1^ ml^−1^ h^−1^. The basic experimental design involved the co-exposure of both GOx and 2.5 mM of INH to HL-60 cells for 4 h. The SILAC experimental design was double standardized; that means the experiment was designed as forward reaction and reverse reaction [2]. For forward reaction, isotope labeled HL-60 cells were treated with both GOx and 2.5 mM of INH for 4 h (treatment group), and non-isotopic HL-60 cells were treated with GOx only for 4 h (control group). In the case of reverse reaction, isotope labeled cells was used as the control group (GOx treatment for 4 h) whereas non-isotopic cells were used as the treatment group (INH and GOx treatment for 4 h).

### 4.2 SILAC cell culture

**Fig. 1:**
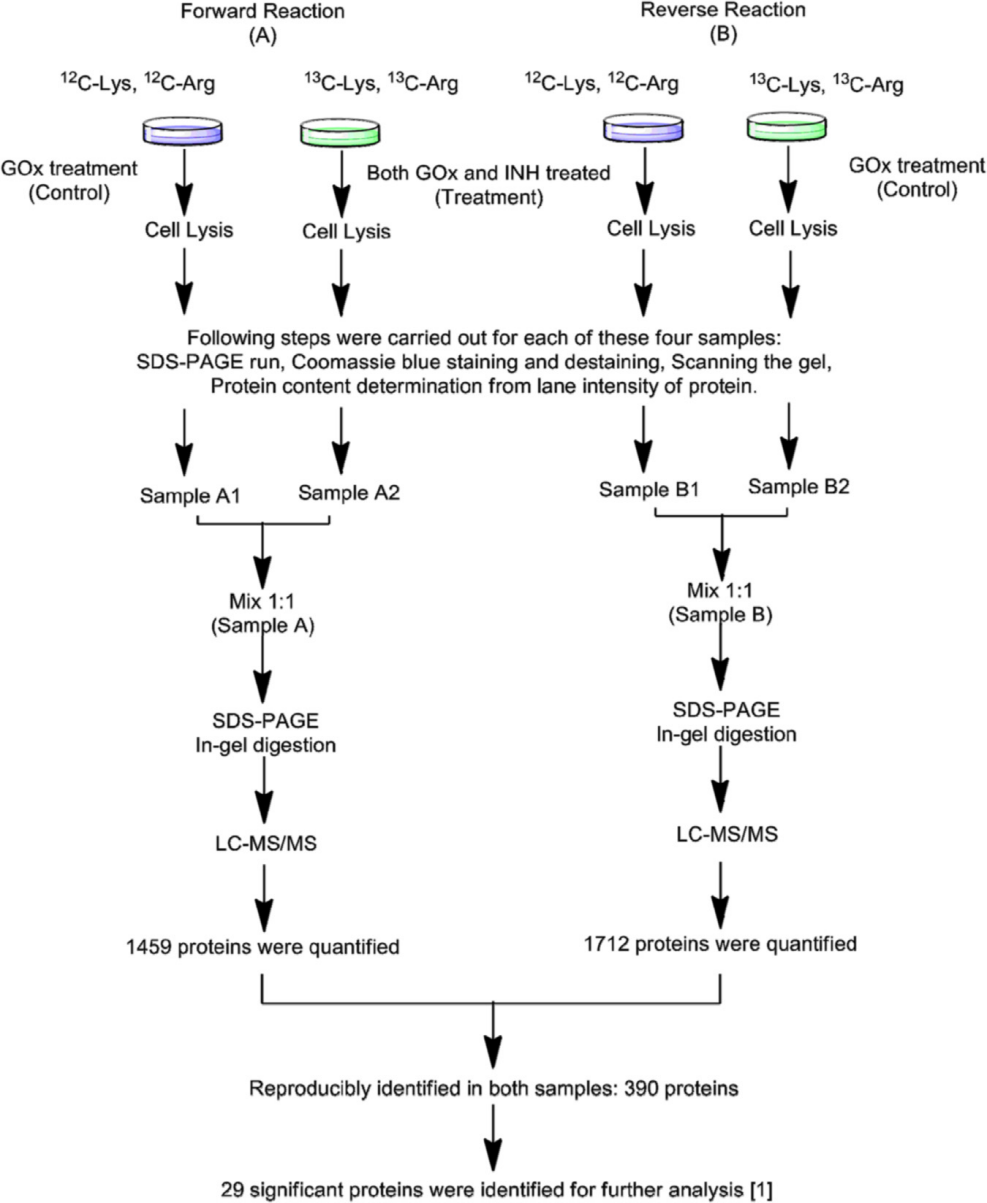
The sequence of steps used (SILAC methodology) for quantitative global proteomics.

HL-60 cells (human promyelocytic leukemia cells, purchased from ATCC, Manassas, VA) were divided into two: one is cultured in “heavy media” and another is cultured in “light media”. Both media were prepared by using “base RPMI-1640 medium” (purchased from Sigma Chemical Co.) which did not contain L-lysine, L-arginine and L-leucine. L-leucine was added to this base RPMI-1640 to a final concentration of 0.05 mg/mL. 10% dialyzed fetal bovine serum (FBS), obtained from Invitrogen, was also added to base medium. Afterwards, the base medium was equally divided into two parts to make them either heavy or light medium. In heavy medium, isotope labeled ^13^C_6_-L-Lysine and^13^C_6_-L-Arginine was added to one part of base medium as 0.04 mg/mL and 0.2 mg/mL, respectively. In light medium, non-isotope labeled ^12^C_6_-L-Lysine and^12^C_6_-L-Arginine was added to the same final concentrations as the isotopically heavy labeled media. Equal amounts of HL-60 cells were transferred into both media and were cultured separately under a humidified atmosphere with 5% CO_2_ at 37 °C for at least 7 passages. Medium was renewed in every 2–3 days depending on cell density [1]. The overall procedure has been shown in Fig. 1.

### 4.3 Cell lysis and protein equalization

The reactions were run for 4 h followed by cell lysis by using RIPA (0.05 g sodium deoxycholate, 100 μL of Triton X-100, and 0.01 g of SDS in 10 mL of PBS). A protease inhibitor cocktail was freshly pre-added to RIPA as 10 µl/ml. Afterwards, 30 µl of each sample was run in different lanes of an SDS-PAGE. The SDS gel was stained by Coomassie blue followed by destaining overnight by using destaining solution (H_2_O: Methanol: Acetic acid, 50:40:10). The gel was scanned through a LI-COR Odyssey gel scanner to quantify the intensity of protein content in each lane. It provided a quantitative check of the ratio of protein between control and treatment of each reaction (A1 and A2, B1 and B2). By using this ratio, the final sample for each pair was prepared by mixing them as a 1:1 ratio. Sample A contained sample-A1 and sample-A2 in 1:1 ratio whereas sample B contained sample-B1 and sample-B2 in 1:1 ratio [1].

### 4.4 SDS-PAGE and in-gel digestion

Both sample A and sample B were run for final SDS-PAGE. 2× loading buffer (0.5 M Tris–HCl pH 6.8, 10% SDS, 1.5% Bromophenol Blue, 20% Glycerol and 5% β-mercaptoethanol) was used for both samples followed by heating to 95 °C for 5 min and then resolved on a 1.0 mm thick 10% polyacrylamide gel. After electrophoresis, the SDS gel was stained with Coomassie Brilliant Blue followed by destained using destaining solution (previously mentioned). It visualized the bands and lanes of SDS gel. Each lane was excised from gel and placed on a gel digestion plate where each lane was again excised into 12 equal pieces. 100 mM NH_4_HCO_3_/acetonitrile (50:50) was used for further de-stained. The reduction and alkylation reactions were then carried out by using reducing solution (10 mM β-mercaptoethanol, 100 mM NH_4_HCO_3_) and alkylating solution (55 mM iodoacetamide in 100 mM NH_4_HCO_3_), followed by dehydration. In-gel digestion was conducted by using trypsin (6 ng/uL) overnight (~16 h). Two steps extraction procedure was then carried out to extract the tryptic peptides from the gel (step-1: extraction by 97% water/2% acetonitrile/1% formic acid solution; step-2: extraction by 1:1 extraction buffer and acetonitrile) [1], [3].

### 4.5 LC–MS/MS analysis

Tryptic peptides were resolved in 25% acetonitrile and 1% v/v formic acid for LC–MS/MS. The nanoflow HPLC (Easy-nLC II, Thermo Scientific) coupled to the LTQ XL-Orbitrap hybrid mass spectrometer (MS) (Thermo Scientific) was used here. A picofrit fused silica capillary column (ProteoPepII, C18) with 100 μm inner diameter (300 Å 5 μm, New Objective) was utilized for nanoflow chromatography and electrospray ionization. The injection flow rate of peptide mixtures was kept at 3000 nL/min, and the resolved rate was kept at 500 nL/min using 60 min linear acetonitrile gradients from 0% to 45% v/v aqueous acetonitrile in 0.2% v/v formic acid.

The data-dependent acquisition mode of the mass spectrometer was used to record high accuracy and high-resolution survey orbitrap spectra by using external mass calibration, with a resolution of 60,000 and *m*/*z* range of 400–2000. It sequentially fragmented the ten most intense multiply charged ions by using collision induced dissociation. The spectra of their fragments were then recorded in the linear ion trap. However, two fragmentations of all precursors selected for dissociation were dynamically excluded for 60 s. Proteome Discoverer 1.3 (Thermo Scientific) and the non-redundant reviewed human database from Uniprot were used for Data processing. A precursor mass tolerance of 10 ppm and a fragment mass tolerance of 0.8 Da were used as search parameters. Peptides were searched with carbamidomethyl cysteine as a static modification and oxidized methionine, ^13^C-lysine and ^13^C-arginine as dynamic modifications [1], [3].

### 4.6 Data processing

**Fig. 2:**
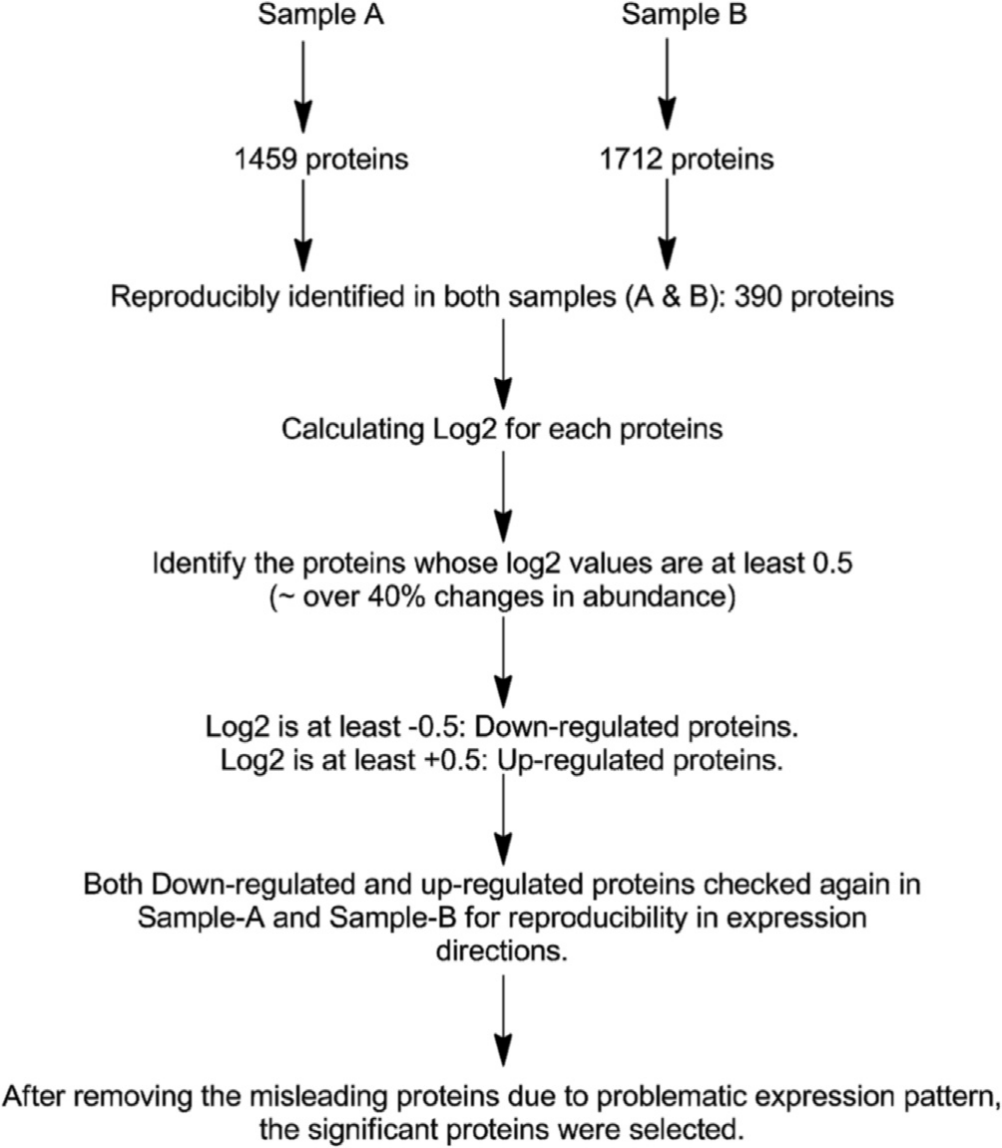
Data processing step used to acquire SILAC data and identify significant proteins.

Proteome Discoverer 1.3 software (Thermo Scientific) was used to extract only the high confidence peptide MS data into a Microsoft Excel file which contained protein accession number as well as their expression level expressed as ^13^C/^12^C ratios. At least two high confidence peptides were used as threshold value to judge the certainty of protein identification. A total of 1459 and 1712 proteins were identified in samples A and B, respectively. Proteins those were reproducibly identified in both samples (390 proteins) were taken for further analysis to find the significant protein expression change by calculating log2 values for all proteins (390 proteins, described above). The steps of data processing have been illustrated in Fig. 2.

## Footnotes

Appendix A Supplementary data associated with this article can be found in the online version at doi:10.1016/j.dib.2016.01.035.

## References

[1] Khan Saifur R., Aljuhani Naif, Morgan Andrew G.M., Baghdasarian Argishti, Fahlman Richard P., Siraki Arno G. (2015). Cytoprotective effect of isoniazid against H_2_O_2_ derived injury in HL-60 cells. Chem.-Biol. Interact..

[2] (2014). Current status and future prospects of toxicogenomics in drug discovery. Drug Discov. Tod..

[3] Khan S.R., Baghdasarian A., Nagar P.H., Fahlman R., Jurasz P., Michail K., Siraki Arno G. (2015). Proteomic profile of aminoglutethimide-induced apoptosis in HL-60 cells: role of myeloperoxidase and arylamine free radicals. Chem.-Biol. Interact..

